# 6-*O*-*trans*-feruloyl Catalpol, a Natural Antioxidant from the Stem Bark of *Catalpa ovata*, Accelerates Liver Regeneration *In Vivo* via Activation of Hepatocyte Proliferation Signaling Pathways

**DOI:** 10.3390/antiox14101210

**Published:** 2025-10-06

**Authors:** Jiyoung Park, Yun-Seo Kil, Ho Jin Yi, Eun Kyoung Seo, Hyun Ae Woo

**Affiliations:** 1 College of Pharmacy, Graduate School of Pharmaceutical Sciences, Ewha Womans University, Seoul 03760, Republic of Korea; jypark89@ewhain.net; 2 Fluorescence Core Imaging Center, Department of Life Science, Ewha Womans University, Seoul 03760, Republic of Korea; 3 College of Pharmacy and Inje Institute of Pharmaceutical Sciences and Research, Inje University, Gimhae 50834, Gyeongnam, Republic of Korea; yskil@inje.ac.kr; 4 College of Pharmacy, Graduate School of Applied Science and Technology for Skin Health and Aesthetics, Ewha Womans University, Seoul 03760, Republic of Korea; yihoz@ewhain.net

**Keywords:** 6-*O-trans*-feruloyl catalpol, liver regeneration, partial hepatectomy, hepatocyte proliferation signaling, *Catalpa ovata*, natural product-derived therapeutics

## Abstract

Background: Liver regeneration is a complex process involving multiple signaling pathways that coordinate hepatocyte proliferation, survival, and tissue repair. Natural compounds like silymarin, ursolic acid, quercetin, and resveratrol have shown regenerative potential, though their precise molecular mechanisms remain unclear. 6-*O-trans*-feruloyl catalpol (6FC), a major bioactive compound from *Catalpa ovata*, exhibits anti-inflammatory and potential antioxidant effects via regulation of NF-κB signaling and redox-sensitive pathways such as Akt and MAPK, which are critical for cell survival and proliferation. Moreover, 6FC exhibits peroxynitrite-scavenging activity, suggesting its potential antioxidant properties that may protect hepatocytes from oxidative damage during regeneration. However, the role of 6FC in liver regeneration has not been elucidated, positioning it as a promising natural therapeutic candidate for hepatic repair. Purpose: This study aimed to determine whether 6FC promotes hepatocyte proliferation and liver regeneration *in vivo* using a 2/3 PHx mouse model, and to validate its proliferative effects *in vitro* with HGF-stimulated Hep3B cells. Methods: A 2/3 PHx liver regeneration model was used to evaluate 6FC-mediated liver regeneration. Histological and molecular analyses assessed hepatocyte proliferation and signaling activation. HGF-stimulated Hep3B cells were also used to examine 6FC proliferative effects *in vitro*. Results: 6FC significantly promoted liver regeneration by restoring the liver-to-body weight ratio and reducing serum ALT and AST levels without inducing excessive immune responses. Mechanistic studies revealed that 6FC activates Akt and MAPK pathways, increases the expression of critical growth factors, and upregulates cell cycle regulators. These effects were also observed in HGF-stimulated Hep3B cells, suggesting that 6FC may enhance hepatocyte proliferation without triggering excessive immune responses. Conclusions: 6FC accelerates hepatocyte proliferation and promotes liver regeneration by activating key redox-sensitive signaling pathways, highlighting its potential as a natural antioxidant-based therapeutic agent.

## 1. Introduction

Hepatocytes in the liver are typically quiescent under normal physiological conditions, maintaining a stable, non-proliferative state. However, upon tissue injury, such as partial hepatectomy (PHx), these cells can re-enter the cell cycle, leading to rapid mitotic expansion to restore lost tissue [1,2,3]. PHx is a widely used experimental model to study liver regeneration, as it effectively stimulates compensatory hepatocyte proliferation and functional recovery of the liver. During liver regeneration, quiescent hepatocytes essentially need to be primed to respond to growth factors and promote compensation mechanism of proliferation for the resected liver. However, a substantial number of patients lack sufficient regenerative capacity post-hepatectomy, highlighting the need for therapeutic strategies that enhance hepatocyte proliferation [4,5,6]. Various natural compounds, including silymarin, ursolic acid, quercetin, and resveratrol, have been extensively studied for their ability to promote liver regeneration due to their high efficiency and low toxicity [7,8,9,10,11]. These natural compounds have been studied for their potential to promote liver regeneration due to their relatively low toxicity, although their precise mechanisms of action remain unclear. 6-*O-trans*-feruloyl catalpol (6FC), a major bioactive compound isolated from the stem bark of *Catalpa ovata* (Bignoniaceae), has demonstrated potent anti-inflammatory and peroxynitrite-scavenging activity, suggesting its potential antioxidant properties [12]. *C. ovata* is one of the iridoid-producing plants that has been used as herbal anti-inflammatory remedies [13,14,15,16]. Several studies suggest that its anti-inflammatory effects are mediated primarily through the NF-κB pathway, a key regulator of cell survival, proliferation, and tissue repair. While NF-κB activation may induce apoptosis via cooperation with AP-1 in certain contexts [17], it can also promote anti-apoptotic and regenerative effects in hepatocytes by activating redox-sensitive survival pathways such as Akt and MAPKs [18,19,20]. These signaling cascades are tightly regulated by the cellular redox environment and are central to hepatocyte proliferation and liver regeneration [21,22]. Oxidative stress is a critical modulator of liver regeneration, as excessive ROS generation can impair cell survival, while balanced redox signaling is essential for initiating regeneration-associated gene expression. Liver regeneration is orchestrated by a network of signaling pathways, many of which are redox-sensitive and tightly regulated during the post-hepatectomy response. To understand the molecular events underlying this process, it is essential to analyze the temporal expression patterns of key genes involved in regeneration. Among the key transcription factors, NF-κB plays a pivotal role in regulating hepatocyte proliferation, tissue repair, cell adhesion, and antioxidant responses [23,24,25]. Numerous studies have clearly established that NF-κB activation is crucial for liver regeneration after 2/3 partial hepatectomy (PHx) [26,27,28,29]. Once activated, NF-κB translocates to the nucleus and induces the transcription of genes associated with cell cycle progression, proliferation, and survival [30,31,32]. These findings highlight the central role of NF-κB as a redox-sensitive transcriptional regulator in liver regeneration. Based on these findings, we hypothesized that 6FC promotes liver regeneration by activating redox-sensitive signaling pathways such as NF-κB, Akt, and MAPK. Therefore, this study aimed to determine whether 6FC enhances hepatocyte proliferation and liver regeneration *in vivo* using a 2/3 PHx mouse model, and to validate its proliferative effects *in vitro* with HGF-stimulated Hep3B cells.

## 2. Matrrials and Methods

### 2.1. Animals and Drug Administration

Male C57BL/6J mice, 10 weeks of age (Jackson Laboratory, Bar Harbor, ME, USA), were employed in this study. All procedures were conducted in compliance with the guidelines of the Institutional Animal Care and Use Committee of Ewha Womans University (protocol no. EWHA IACUC 19-001). Animals were maintained under controlled environmental conditions (constant temperature, 12 h light/12 h dark cycle). Experimental groups received oral gavage of either 6FC or silymarin (Sm, 10 mg/mL) once daily for seven consecutive days prior to partial hepatectomy (PHx), and treatment was continued until sacrifice. For the sham-operated groups, drug administration was performed in the same manner as in the experimental groups, but without PHx. Vehicle-treated controls received PBS in place of the compounds and underwent PHx. Positive control mice were treated with Sm under identical conditions.

### 2.2. Surgical Procedures

All surgeries were carried out by a single operator using a consistent and standardized technique. Partial hepatectomy (PHx) was employed as an experimental model to investigate liver regeneration and surgery-induced stress [33,34]. Following anesthesia, approximately two-thirds of the liver, including the median and left lateral lobes, was resected, and regeneration was examined at different time points in separate sets of experiments. Both blood and liver tissues were harvested. Blood samples were centrifuged at 3000× *g* for 5 min to collect serum which was stored at −80 °C.

### 2.3. Biochemical Assays

Blood was obtained from the inferior vena cava, and plasma was isolated by centrifugation at 3000 rpm for 15 min at 4 °C. Plasma alanine aminotransferase (ALT) and aspartate transaminase (AST) levels were determined with the EnzyChrom™ assay kit (BioAssay Systems, Hayward, CA, USA) following the manufacturer’s instructions.

### 2.4. RNA Isolation

Tissue samples were homogenized in 1 mL TRIzol reagent (Invitrogen, Carlsbad, CA, USA) on ice, followed by the addition of 200 μL chloroform. The mixture was vortexed vigorously for 15 sec and allowed to stand for 3 min to facilitate phase separation, then centrifuged at 12,000× *g*, 4 °C for 15 min. After centrifugation, the lysate separated into three phases, and the upper aqueous layer containing RNA was carefully transferred to a fresh tube. An equal volume of 500 μL isopropanol was added, the tube was gently inverted four times, and the mixture was stored at −80 °C for 16 h to precipitate RNA. The frozen mixture was thawed on ice and centrifuged at 12,000× *g* at 4 °C for 10 min. The resulting pellet was washed twice with 70% ethanol prepared in RNase-free water, centrifuged again at 12,000× *g*, 4 °C for 10 min, then dried. The RNA pellet was dissolved in RNase-free water. RNA concentration and purity were assessed with a NanoDrop ND-1000 spectrophotometer (Daemyung, Republic of Korea).

### 2.5. Reverse Transcription Polymerase Chain Reaction (RT-PCR) and Quantitative Real Time PCR (qPCR)

Isolated RNA was used for reverse transcription to synthesize complementary DNA (cDNA) from single-stranded RNA. Two micrograms of RNA were diluted in diethyl pyro carbonate (DEPC)-treated water to a final volume of 20 μL and mixed with a cDNA premix (ECODRY, Kansas City, MO, USA) containing reverse transcriptase, dNTPs, random hexamer primers, and buffer. The reaction mixture was incubated at 42 °C for 60 min for reverse transcription, followed by heating at 70 °C for 10 min to inactivate the reverse transcriptase. The synthesized cDNA was subsequently analyzed by qPCR using the ABI 7300 Real-Time PCR System (Applied Biosystems, Foster City, CA, USA). Each qPCR reaction was performed in a total volume of 20 μL containing 2 μL (40 ng) of cDNA, 10 μL of SYBR Green premix (BIOLINE, London, UK), 0.25 pM of each forward and reverse primers, and autoclaved distilled water. Expression data were normalized to the levels of GAPDH mRNA. The sequences of primers used are provided in Table 1.

### 2.6. Immunoblot Analysis

Cells and tissues were homogenized in ice-cold lysis buffer (20 mM HEPES pH 7.0, 0.15 M NaCl, 10% glycerol, 1% Triton X-100, 1 mM EDTA, 1 mM EGTA, 10 mM β-phosphoglycerate, 1 mM Na_3_VO_4_, 5 mM NaF, 1 μg/mL aprotinin, 1 μg/mL leupeptin, 100 μM PMSF) using either a Polytron homogenizer or sonicator. The homogenates were centrifuged at 15,000 rpm, 4 °C for 15 min. Protein concentrations in the supernatants were measured with the Bradford assay (Bio-Rad, Hercules, CA, USA). Equal amounts of protein were then mixed with sample buffer (62.5 mM Tris-HCl pH 6.8, 10% glycerol, 2% sodium dodecyl sulfate, 0.0125% bromophenol blue, 2.5% β-mercaptoethanol) and heated at 95 °C for 5 min. Proteins were resolved by SDS–polyacrylamide gel electrophoresis (SDS-PAGE) using running buffer (3 g/L Tris, 14.35 g/L glycine, 1 g/L SDS). Separated proteins were transferred onto methanol-activated PVDF membranes (0.45 μm pore size, Millipore, Darmstadt, Germany) using transfer buffer (3.03 g/L Tris, 14.17 g/L glycine, 20% methanol Membranes were blocked with 5% bovine serum albumin (BSA) in Tris-buffered saline with 0.1% Tween-20 (TBST) for 20 min at room temperature on a rocker, and then incubated overnight at 4 °C with primary antibodies diluted 1:2000. Antigen–antibody complexes were detected using HRP-conjugated secondary antibodies (Bio-Rad, Hercules, CA, USA) and visualized with enhanced chemiluminescence reagents (Ab Frontier, Daejeon, Republic of Korea) on the IQ800 system (GE Healthcare, Stockholm, Sweden). The abundance of target proteins was quantitated by densitometric analysis of immunoblots. Bradford assay data were acquired using a SpectraMax M2 Microplate Reader (Molecular Devices, San Jose, CA, USA) at the Fluorescence Core Imaging Center, Ewha Womans University. The primary antibodies used in this study included p-IKKα (#2859s), p-IKBα (#2895S), p-NFκB p65 (Ser536) (#3033_ 93H1), NFκB p65 (#8242_ D14E12), p-AKT (Ser473) (#9271), p-ERK (#9101), ERK (#9102), p-JNK (#9251), p-p38 (#9211s), p-Stat3 (#9145_D3A7) and Stat3 (#4904_79D7) were purchased from CST (Cell Signaling Technology, Danvers, MA, USA). Cyclin D (#SC-717_C-20), cyclin E (#SC-418_M-20), c-myc (#sc_40), GAPDH (#sc_25778), Ikbα (#sc-371), AKT (SC-8312_H-136), JNK (#sc-7345), p38 (sc_7972) were purchased from Santa Cruz Biotechnology. Haptoglobin (#PA5-24174) and Fibrinogen (710386) were purchased from Sigma-Aldrich (St. Louis, MO, USA).

### 2.7. Cell Culture

Hep3B human hepatocellular carcinoma cells were purchased from the American Type Culture Collection (ATCC, Manassas, VA, USA). Cells were cultured in DMEM supplemented with 10% fetal bovine serum (HyClone) and 1% penicillin–streptomycin. Cultures were maintained at 37 °C in a humidified incubator with 5% CO_2_. Subculturing was performed at approximately 80% confluence in accordance with the supplier’s instructions. Cell morphology was routinely monitored under a light microscope. For stimulation experiments, Hep3B cells (1 × 10^6^ per mL) were serum-starved for 16 h and subsequently treated with HGF (10 ng/mL) for the indicated time periods.

### 2.8. Cell Proliferation Assay

For proliferation analysis, cell numbers were determined in monolayer cultures grown in 12-well plates using a hemocytometer. Hep3B cells were seeded at an initial density of 0.5 × 10^5^ cells/mL in medium containing either DMSO (control) or 10 μM of the test compounds. Cells were harvested by trypsinization, and viable cell numbers were counted in triplicate for each group at 24 h intervals. Data are presented as the mean values from three independent experiments. To examine the dose-dependent effects of the compounds on proliferation, Hep3B cells were seeded at an initial density of 0.5 × 10^5^ cells/mL in medium containing the compounds (0.5–10 μM) or DMSO (control). Cell growth was assessed on day 4 by counting cell numbers. Cell counts were performed in triplicate for each group, and all experiments were repeated independently three times.

### 2.9. Statistical Analysis

All quantitative data are expressed as the mean ± standard deviation (SD) from at least three independent experiments. Statistical analyses were performed using Student’s *t*-test with SigmaPlot software (version 10.0; Systat Software, San Jose, CA, USA). A *p*-value < 0.05 was considered statistically significant.

### 2.10. Materials

6FC was isolated from the stem bark of *Catalpa ovata* G. Don. (Bignoniaceae), as described previously [12]. The purity evaluation was performed by analytical HPLC (purity > 99%). Sm was purchased from Sigma (St. Louis, MO, USA) (Supplementary Materials).

## 3. Results

### 3.1. 6FC Promotes Liver Regeneration After 2/3 PHx in Mice

**Figure 1:**
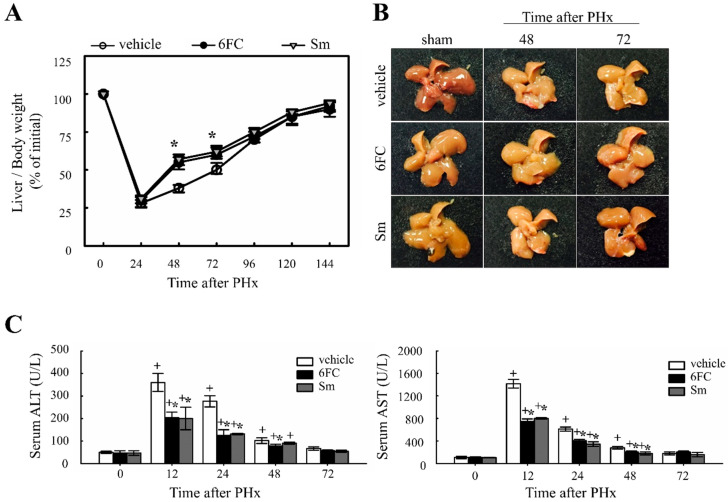
Effects of 6-*O*-*trans*-feruloyl catalpol on liver regeneration and liver injury in mice after 2/3 partial hepatectomy. (**A**,**B**) Effect of 6-*O*-*trans*-feruloyl catalpol on liver-to-body weight ratio over time. Mice were orally administered 6-*O-trans*-feruloyl catalpol, positive control (p.c., silymarin, or vehicle once daily for seven days prior to surgery, followed by 2/3 partial hepatectomy. Liver/body weight ratios were measured at different time points post-partial hepatectomy to assess liver regrowth. (**C**) Effect of 6-*O-trans*-feruloyl catalpol on serum alanine aminotransferase and aspartate aminotransferase levels after partial hepatectomy. The levels of alanine aminotransferase and aspartate aminotransferase were quantified to evaluate hepatic injury. Data are presented as the mean ± SD (*n* ≥ 5). + *p* < 0.05 vs. sham-operated group. * *p* < 0.05 vs. vehicle-treated group.

To evaluate the effect of 6FC on liver regeneration, the liver-to-body weight ratio was measured. Previous studies have reported that Sm accelerates hepatocyte proliferation by enhancing protein synthesis, thereby promoting liver regeneration [35,36]. In this study, Sm was used as a positive control. The experimental group received oral administration of 6FC daily for seven days before surgery, which continued until sacrifice. No significant differences in dietary intake were observed among the experimental groups throughout the study period. As shown in Figure 1A,B, the liver/body weight ratio in the 6FC-treated group increased significantly at 48–72 h after PHx compared with the vehicle group, showing efficacy comparable to the Sm-treated group. Notably, 6FC did not induce excessive liver enlargement even after 14 days post-PHx, indicating that it promotes regeneration without causing abnormal liver overgrowth. Additionally, PHx significantly increased serum ALT and AST levels compared to the sham-operated group, indicating liver injury (Figure 1C). However, pretreatment with 6FC (10 mg/kg) significantly reduced ALT and AST levels, suggesting that 6FC may support liver recovery without inducing excessive immune responses. Unlike conventional natural compounds that primarily focus on reducing inflammation, 6FC appears to restore liver function without triggering excessive immune activation, providing a more stable therapeutic approach. This reduction was comparable to that observed in the Sm-treated group, which served as a positive control. Furthermore, these results indicate that 6FC suppresses excessive inflammatory responses. These findings suggest that 6FC facilitates liver regeneration following PHx, restoring liver mass without causing excessive growth or damage.

### 3.2. 6FC Activates Cell Cycle Regulatory Proteins After 2/3 PHx in Mice

**Figure 2:**
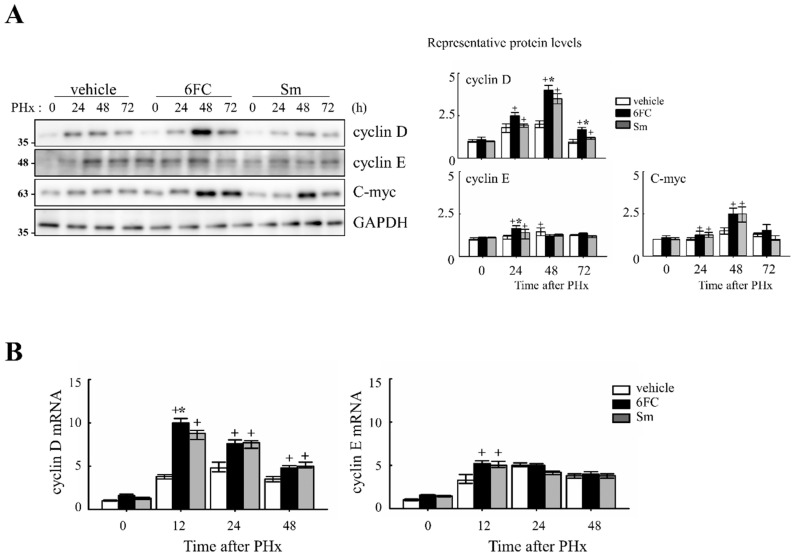
Effects of 6-*O*-*trans*-feruloyl catalpol on the expression of cell cycle genes in mice after 2/3 partial hepatectomy. (**A**) 6-*O-trans*-feruloyl catalpol enhances the expression of cell cycle-related proteins. Representative immunoblots showing the expression levels of cyclin D, cyclin E, and c-myc in liver tissues after partial hepatectomy. Densitometric analysis was performed to quantify the relative expression levels of these proteins using Image J software (version 1.53, National Institutes of Health, Bethesda, MD, USA). (**B**) Effects of 6-*O-trans*-feruloyl catalpol on the mRNA levels of cyclin D and cyclin E. Real-time PCR analysis was conducted to evaluate the expression of cyclin D1 and cyclin E mRNA in liver tissues post-partial hepatectomy. Data are presented as the mean ± SD (*n* ≥ 5). + *p* < 0.05 vs. vehicle-treated group. * *p* < 0.05 vs. silymarin -treated group.

To determine whether 6FC accelerates the cell cycle during liver regeneration, we analyzed the expression of cell cycle-related proteins in the liver. As shown in Figure 2A, 6FC significantly increased the protein levels of cyclin D and cyclin E at 24 and 48 h post-PHx, compared to both the sham-operated and vehicle-treated groups. A similar increase was observed in the Sm-treated group, indicating that 6FC exerts a comparable effect on cell cycle progression.

At the mRNA level, *cyclin D1* and *cyclin E* expressions were similar between the vehicle- and 6FC-treated groups before PHx but were significantly upregulated in the 6FC-treated group at 24 h post-PHx (Figure 2B). In addition, *c-myc* expression showed a similar pattern of upregulation (Figure 2A), further supporting the role of 6FC in promoting liver regeneration. During liver regeneration, the sequential and regulated activation of proto-oncogenes such as *c-myc* is a key event. These genes serve as markers of the initial phase of liver regeneration, with *c-myc* activation being a characteristic feature of early growth in most cell systems [37,38,39]. The observed upregulation of *c-myc* suggests that 6FC may support early hepatocyte proliferation during liver regeneration by promoting early-phase cell cycle progression. These results suggest that 6FC may contribute to liver regeneration by promoting the timely activation of cell cycle regulators, potentially enhancing tissue recovery while preserving cellular homeostasis.

### 3.3. 6FC Modulates NF-κB and STAT3 Pathways During Liver Regeneration

NF-κB is rapidly activated following hepatic injury and plays a pivotal role in promoting hepatocyte survival and regeneration by regulating anti-apoptotic and proliferative genes [12,40].

**Figure 3:**
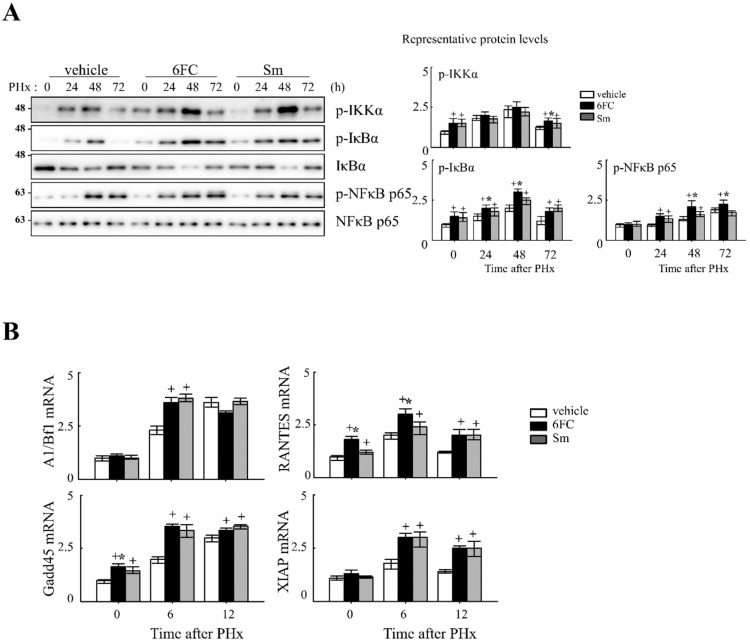
6-*O-trans*-feruloyl catalpol promotes the activation of the NF-κB signaling pathway in mice after 2/3 Partial hepatectomy. (**A**) Effect of 6FC on NF-κB-related protein levels. Representative immunoblots show the expression levels of p-IKKα, p-IκBα, and p-NF-κB p65 in liver tissues post-Partial hepatectomy. Densitometric analysis was performed using ImageJ software to quantify the relative expression levels of these proteins. (**B**) Effects of 6-*O-trans*-feruloyl catalpol on the mRNA levels of NF-κB downstream target genes. Real-time PCR analysis was conducted to evaluate the expression of NF-κB-regulated genes in liver tissues after Partial hepatectomy. Data are presented as the mean ± SD (*n* ≥ 5). + *p* < 0.05 vs. vehicle-treated group. * *p* < 0.05 vs. silymarin-treated group.

Previously, 6FC has been reported to activate the NF-κB signaling pathway. In this study, we aimed to determine whether the same activation occurs during liver regeneration following PHx by analyzing the expression of key upstream regulators and downstream target genes. NF-κB is rapidly activated after partial hepatectomy and functions as a pro-survival checkpoint that prevents hepatocyte apoptosis and ensures timely regenerative responses. Both hepatocyte and Kupffer-cell NF-κB contribute to this effect, as demonstrated by early genetic studies [41,42] and reinforced by recent reviews that emphasize its integration with cytokine and redox signaling pathways during liver repair [6,43,44]. NF-κB activation was assessed by measuring phosphorylation levels of key upstream regulators, including p-IKKα, p-IκBα, and p-NF-κB p65. As shown in Figure 3A, these protein levels were significantly increased in the 6FC-treated group at 24 and 48 h post-PHx, compared to the sham-operated and vehicle-treated groups. Furthermore, NF-κB downstream target gene expression was analyzed by RT-PCR at 0, 6, and 12 h post-PHx. The results indicate that 6FC treatment led to an upregulation of NF-κB target genes during liver regeneration (Figure 3B), suggesting that 6FC enhances NF-κB signaling to support hepatocyte survival and proliferation.

**Figure 4:**
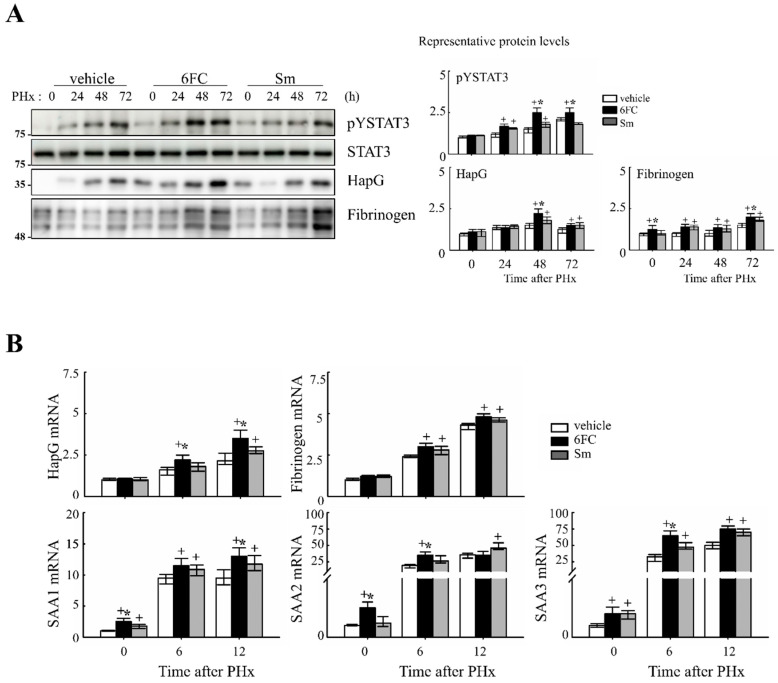
6-*O-trans*-feruloyl catalpol promotes the activation of the STAT3 signaling pathway in mice after 2/3 Partial hepatectomy. (**A**) Effect of 6-*O-trans*-feruloyl catalpol on STAT3 phosphorylation and STAT3 downstream target gene expression. Representative immunoblots show the expression levels of phosphorylated STAT3 (pYSTAT3) and its downstream target proteins. Densitometric analysis was performed using ImageJ software to quantify the relative protein expression. (**B**) Effects of 6-*O-trans*-feruloyl catalpol on the mRNA levels of STAT3 downstream target genes. Real-time PCR analysis was conducted to evaluate the expression of STAT3-regulated genes in liver tissues post-Partial hepatectomy. Data are presented as the mean ± SD (*n* ≥ 5). + *p* < 0.05 vs. vehicle-treated group. * *p* < 0.05 vs. silymarin-treated group.

Similarly, STAT3 is activated by cytokines and growth factors, leading to its dimerization and nuclear translocation, where it binds to target genes and induces transcription [45]. STAT3 plays a critical role in liver regeneration, as demonstrated in previous studies [46,47]. In this study, STAT3 tyrosine phosphorylation (pYSTAT3) was significantly increased in the 6FC-treated group following PHx, compared to the sham-operated and vehicle-treated groups (Figure 4A). Additionally, mRNA levels of STAT3 target genes were higher in the 6FC-treated group, further supporting the activation of this pathway (Figure 4B).

These results suggest that 6FC promotes liver regeneration through coordinated activation of both NF-κB and STAT3 pathways, which are central to redox signaling, hepatocyte survival, and proliferative responses following hepatic injury.

### 3.4. 6FC Enhances Early Gene Expression Involved in Liver Regeneration Following 2/3 PHx in Mice

To further elucidate the molecular mechanisms by which 6FC promotes liver regeneration, we analyzed the mRNA expression of transcription factors and growth factors, which are essential for hepatic regeneration.

**Figure 5:**
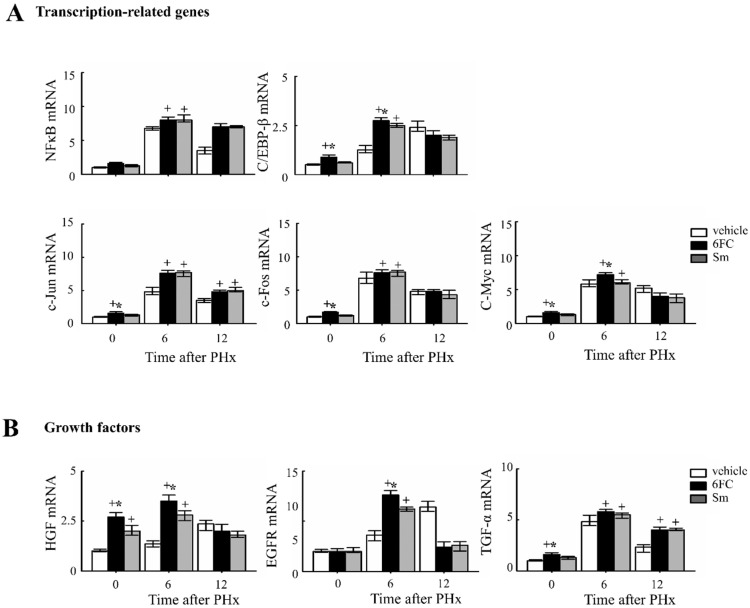
6-*O-trans*-feruloyl catalpol affected the expression of liver regeneration related genes in mice after 2/3 Partial hepatectomy. (**A**) Effects of 6-*O*-*trans*-feruloyl catalpol on the mRNA levels of transcription-related genes. The expression levels of *NF-κB*, *C/EBP-β*, *c-Jun* and *c-Fos* mRNA were measured using Real-time PCR to evaluate their role in liver regeneration. (**B**) Effects of 6-*O*-*trans*-feruloyl catalpol on the mRNA levels of growth factors and transcription factors. Real-time PCR analysis was conducted to assess the expression of growth factors, including *HGF*, *EGFR* and *TGF-β*, which are critical for liver regeneration. Data are presented as the mean ± SD (*n* ≥ 5). + *p* < 0.05 vs. vehicle-treated group. * *p* < 0.05 vs. silymarin-treated group.

Given that STAT3 and NF-κB activation are critical for the priming phase of liver regeneration and contribute to the early response following PHx [48,49,50,51]. As shown in Figure 5A, the 6FC-treated group exhibited significantly higher induction of *c-Jun* and *c-Fos* mRNA expression at 6 h post-PHx compared to the sham-operated and vehicle-treated groups. Since *c-Jun* and *c-Fos* are key components of the activator protein-1 (AP-1) complex, which plays a central role in liver regeneration, these results indicate that 6FC effectively triggers the transcriptional machinery involved in early-phase recovery. Notably, HGF and C/EBP-β levels were also significantly elevated in the livers of sham-operated 6FC-treated mice, suggesting a potential role of 6FC in priming regenerative readiness even in the absence of injury. C/EBP-β activation has been reported to promote *cyclin E* expression and plays a crucial role in hepatic function and regeneration [52,53,54,55]. Similarly, HGF is a well-established mitogenic factor for multiple cell types and has major effects on liver growth [56,57]. HGF is also known to stimulate hepatocyte DNA synthesis, influencing the balance between cell proliferation and apoptosis during liver regeneration [58,59]. Collectively, these findings suggest that 6FC promotes early regenerative signaling through transcriptional upregulation of regeneration-associated factors, contributing to efficient tissue recovery and hepatic homeostasis, potentially through modulation of redox-sensitive signaling networks.

### 3.5. 6FC Activates Redox-Sensitive Akt and MAPK Signaling Pathways During Liver Regeneration

**Figure 6:**
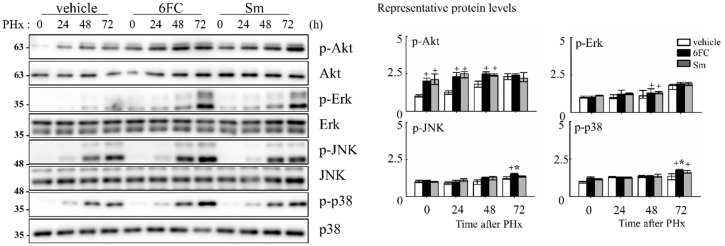
6-*O*-*trans*-feruloyl catalpol regulates redox-sensitive Akt phosphorylation and MAPKs signaling pathway in mice after 2/3 Partial hepatectomy. Effect of 6-*O*-*trans*-feruloyl catalpol on Akt phosphorylation and MAPK signaling proteins. Representative immunoblots show the expression levels of phosphorylated Akt (p-Akt), Erk, JNK, and p38 in liver tissues after partial hepatectomy. Densitometric analysis was performed to quantify the relative levels of phosphorylated Akt and MAPK family proteins using Image J software. Data are presented as the mean ± SD (*n* ≥ 5). + *p* < 0.05 vs. vehicle-treated group. * *p* < 0.05 vs. silymarin-treated group.

To elucidate the intracellular signaling events underlying 6FC-mediated hepatoprotection and regeneration, we assessed the activation status of survival-associated pathways during the early phase of liver regeneration. Akt, a serine/threonine kinase, is a key effector in the phosphatidylinositol 3-kinase (PI3K)/Akt signaling cascade and is known to promote cell survival by inhibiting apoptosis in response to mitogenic stimuli [60,61,62]. In this study, we observed that phosphorylated Akt (p-Akt) levels were sustained in the 6FC-treated group both before and after PHx, indicating prolonged Akt activation (Figure 6). This trend was also observed in the Sm-treated group, suggesting comparable effects on Akt signaling. Given the role of Akt in modulating downstream effectors, we next evaluated the phosphorylation of mitogen-activated protein kinases (MAPKs), including Erk, JNK, and p38, which are essential mediators of redox-sensitive signal transduction and liver regeneration [63,64]. As shown in Figure 6, 6FC markedly enhanced the phosphorylation of all three MAPK family members following PHx. These pathways are also involved in HGF-induced NF-κB activation through canonical IκB degradation and MAPK-dependent transcriptional activation [65,66], further linking redox-regulated kinase signaling to regenerative outcomes. These findings suggest that 6FC promotes liver regeneration by sustaining Akt and MAPK signaling pathways, which are crucial for hepatocyte survival and proliferation. Importantly, this activation occurred without evidence of excessive immune response, highlighting 6FC’s potential as a redox-active compound that balances regeneration with immune tolerance.

### 3.6. 6FC Promotes Hepatocyte Proliferation In Vitro Through Redox-Sensitive Signaling Pathways

To investigate whether 6FC enhances hepatocyte proliferation through redox-sensitive pathways, we conducted *in vitro* experiments using human hepatoma Hep3B cells. Since MAPK cascades act as important intermediates in HGF-induced NF-κB activation, we hypothesized that 6FC may enhance HGF-mediated proliferative signaling.

**Figure 7:**
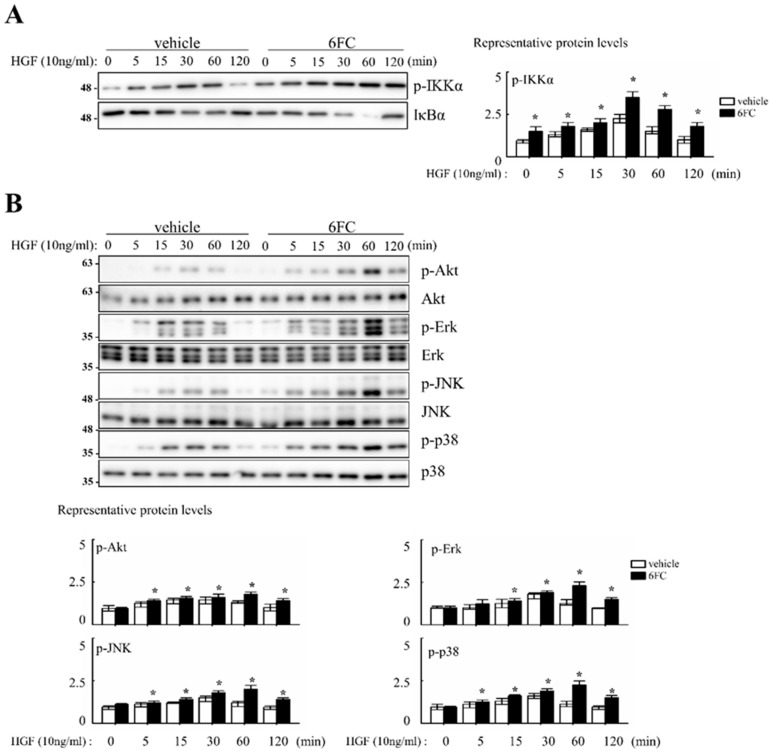
6-*O-trans*-feruloyl catalpol regulates NF-κB, Akt phosphorylation and MAPKs signaling pathway *in vitro*. (**A**) Effect of 6-*O-trans*-feruloyl catalpol on the levels of the NF-κB related proteins. (**B**) Effect of 6-*O-trans*-feruloyl catalpol on the levels of Akt phosphorylation and MAPKs signaling proteins. Densitometric analysis of relative NF-κB related proteins, Akt phosphorylation and MAPKs in immunoblots. The intensity of immunoblots bands was quantitated using Image J software Data are presented as the mean ± SD (*n* ≥ 4). * p< 0.05 vs. vehicle.

Cell proliferation was evaluated following treatment with 10 µM 6FC and 10 ng/mL HGF in complete media. Cell counts were performed every 24 h over a 4-day incubation period. Microscopic observations revealed no differences in cell morphology or attachment between treated and control groups. Compared to the DMSO control, 6FC treatment increased cell numbers by 19%, 76%, 39%, and 37% on days 1–4, respectively. Sm treatment also promoted proliferation, but to a lesser extent (12%, 15%, 7%, and 21%) (Table 2). Cell proliferation was evaluated following treatment with 10 µM 6FC and 10 ng/mL HGF in complete media. Cell counts were performed every 24 h over a 4-day incubation period. Microscopic observations revealed no differences in cell morphology or attachment between treated and control groups. Compared to the DMSO control, 6FC treatment increased cell numbers by 19%, 76%, 39%, and 37% on days 1–4, respectively. Sm treatment also promoted proliferation, but to a lesser extent (12%, 15%, 7%, and 21%) (Table 2). We next assessed the dose-dependent effect of 6FC on hepatocyte proliferation. Hep3B cells were treated with 0.5, 1, 5, or 10 µM of 6FC or Sm. After 4 days, 6FC significantly increased cell numbers in a dose-dependent manner, with proliferation rates of 10.9%, 12.1%, 29.7%, and 41.7%, respectively. Sm also showed a modest increase (4.1%, 7.0%, 6.9%, and 15.5%) (Table 3). To determine whether these proliferative effects were associated with NF-κB signaling, we assessed pathway activation via Western blotting. 6FC pre-treatment significantly increased the phosphorylation of IKKα (p-IKKα), suggesting sustained NF-κB activation. This effect was further enhanced by HGF co-treatment (Figure 7A). Additionally, 6FC promoted sustained phosphorylation of Akt and MAPK pathway components—including p-Akt, p-Erk, p-JNK, and p-p38—without altering total protein levels, indicating that the observed activation was due to post-translational modification rather than increased expression (Figure 7B).

Collectively, these results suggest that 6FC supports hepatocyte proliferation by activating redox-sensitive survival pathways, including NF-κB, Akt, and MAPKs. The observed synergy with HGF further highlights its potential as a regenerative compound. The consistency between *in vitro* and *in vivo* findings supports the role of 6FC as a bioactive molecule with antioxidant-mediated regenerative properties.

## 4. Discussion

The extract of *C. ovata* has been traditionally used for the treatment of inflammatory diseases in East Asia [14]. However, the liver regenerative effects and underlying molecular mechanisms of 6-*O*-*trans* feruloyl catalpol (6FC), the major bioactive compound in *C. ovata*, have not been previously elucidated. In this study, we demonstrate for the first time that 6FC promotes liver regeneration by stimulating hepatocyte proliferation, providing novel mechanistic insights into its regenerative potential. The liver has a unique capacity to regenerate; however, this process is accompanied by oxidative stress and immune activation that may impair efficient regeneration.

Although the extract of *C. ovata* has long been known for its anti-inflammatory properties, the ability of 6FC to promote liver regeneration through hepatocyte proliferation has not been previously established, highlighting the novel contribution of this study. The liver has a unique capacity to regenerate; however, an inflammatory response occurs during resection, in part mediated by Kupffer cells, that influences the speed of regeneration [67,68]. In the current study, we confirmed and extended those findings by demonstrating that *C. ovata* is crucial for the ability of hepatocyte proliferation in the major component 6FC treated group after 2/3 PHx.

Our data show that 6FC restores liver mass without inducing excessive liver enlargement or immune hyperactivation, suggesting a well-balanced regenerative response. 6FC treatment significantly increased the liver-to-body weight ratio following 2/3 PHx and reduced serum ALT and AST levels, reflecting accelerated tissue recovery and attenuated liver injury (Figure 1). ALT and AST are not only markers of hepatocellular necrosis but are also associated with oxidative stress-induced apoptosis [6,69]. Consistent with this, the reduced ALT/AST levels and attenuated macrophage infiltration observed in the 6FC group suggest that 6FC protects hepatocytes from oxidative stress-mediated apoptotic injury, thereby supporting tissue recovery. Recent studies also emphasize the central role of oxidative stress and mitochondrial dysfunction in regulating hepatocyte fate during regeneration [70,71]. In addition, 6FC upregulated the expression of cell cycle regulators such as cyclin D1, cyclin E, and c-myc, which are essential for hepatocyte proliferation during the early regenerative phase (Figure 2). At the signaling level, 6FC activated NF-κB and STAT3, both of which are key regulators of hepatocyte survival, proliferation, and anti-apoptotic responses during liver regeneration (Figure 3 and Figure 4). Furthermore, 6FC markedly enhanced the phosphorylation of redox-sensitive pathways, including Akt and MAPK signaling components—namely Erk, JNK, and p38—further supporting its role in promoting survival and proliferative signaling in regenerating hepatocytes (Figure 6). Because mitochondria are central hubs of ROS generation and redox signaling, the activation of Akt and MAPK pathways by 6FC may reflect preserved mitochondrial function under oxidative stress, thereby enhancing hepatocyte survival and proliferation. *In vitro* assays using Hep3B cells confirmed that 6FC promotes hepatocyte proliferation in a dose-dependent manner and sustains activation of NF-κB, Akt, and MAPKs, particularly when combined with HGF (Figure 7). In addition, peroxisome proliferator-activated receptors (PPARs) are known to regulate lipid and carbohydrate metabolism as well as hepatocyte survival during liver regeneration. Dysregulated PPAR activity under oxidative stress conditions has been linked to impaired energy homeostasis and hepatocyte death. The ability of 6FC to restore proliferative signaling while reducing inflammatory responses suggests that it may also help maintain PPAR activity and cellular energy metabolism, thereby contributing to hepatocyte survival and regenerative capacity [70,71,72]. Currently, hepatic steatosis represents the leading cause of chronic liver injury and has been redefined under the concept of metabolic dysfunction-associated fatty liver disease (MAFLD). Dietary factors play a central role in the development and progression of MAFLD, and accumulating evidence suggests that oxidative stress and impaired energy metabolism contribute to disease pathogenesis. Although our study employed an acute PHx model, the ability of 6FC to reduce oxidative stress, support hepatocyte proliferation, and attenuate inflammatory responses raises the possibility that this compound may also be beneficial in conditions of chronic liver damage such as MAFLD. Future studies using diet-induced fatty liver models will be necessary to validate this therapeutic potential. From a clinical perspective, these findings highlight the potential of 6FC as a safe and effective adjunctive therapy following hepatic resection and possibly in chronic liver diseases characterized by oxidative stress and impaired regeneration.

## 5. Conclusions

Collectively, these findings reveal that 6FC promotes liver regeneration through transcriptional and signaling activation of early regeneration-associated genes and pathways, including cyclins, proto-oncogenes, growth factors, and redox-regulated survival kinases. 6FC, a natural antioxidant from the stem bark of *Catalpa ovata*, may serve as a promising therapeutic agent for enhancing liver regeneration, particularly in conditions of impaired regenerative capacity or oxidative stress-related liver injury, offering a safe and effective adjunctive strategy following hepatic resection.

## Figures and Tables

**Table 1:** Primer sequences of target genes for RT-PCR.

Genes	Forward Primer	Reverse Primer	Accession
*A1*/*Bf1*	TGC CAG GGA AGA TGG CTG AG	TCC GTA GTG TTA CTT GAG GAG	NM_009742.3
*C*/*EBP-β*	CGCCTTTAGACCCATGGAAG	CCCGTAGGCCAGGCAGT	NM_001287738.1
*Cyclin E*	GCAGCGAGCAGGAGACAGA	TGCTTCCACACCACTGTCTTTG	NM_007633.2
*Cyclin D*	CGTGGCCTCTAAGATGAAGGA	TCGGGCCGGATAGAGTTGT	NM_007631.3
*EGFR*	TCAGCAACAACCCCATCCTC	GCTTGGATCACATTTGGGGC	NM_007912.4
*Fibrinogen*	GATGAACAAATGTCACGCAGGCCA	GCCCAAATAATGCCGTCGTCGAAA	NM_133862.2
*c-Fos*	AATGGTGAAGACCGTGTCAGG	CCCTTCGGATTCTCCGTTTCT	NM_010234.3
*Gadd45*	TGAGCTGCTGCTACTGGAGA	GCAGGATCCTTCCATTGTGA	NM_007836.1
*GAPDH*	AGAACATCATCCCTGCATCC	GGTCCTCAGTGTAGCCCAAG	NM_001411841.1
*Haptoglobin*	TATGGATGCCAAAGGCAGCTTTCC	TCGCTGTGGTTCAGGAAGAGGTTT	NM_017370.2
*HGF*	GGTGTATCAGGAACAGGGGC	GTCAAATTCATGGCCAAACCCT	NM_001289461.1
*c-Jun*	TCTTCATTTTCTCACCAACTGCTT	CTCTCCAAATGCTCCCCAAA	NM_010591.2
*c-Myc*	ACTTACAATCTGCGAGCCAGGACA	GCCCAAAGGAAATCCAGCCTTCAA	NM_010849.4
*NF-* *κB*	TGGCAGCTCTTCTCAAAGCA	CCAAGAGTCGTCCAGGTCATAGA	NM_001410442.1
*RATENS*	CAGCAGCAAGTGCTCCAATCTT	TTCTTGAACCCACTTCTTCTCTGG	NM_013653.3
*SAA1*	GCGAGCCTACTCTGACATGAAG	CCCCCGAGCATGGAAGTATT	NM_001163892.2
*SAA2*	AAGCTGGCTGGAAAGATGGA	CCTTTGGGCAGCATCATAGTTC	NM_011314.3
*SAA3*	CCTGGGCTGCTAAAGTCATCA	CCATGTCCCGTGAACTTCTGA	NM_011315.3
*TGF-* *β*	CCGCAACAACGCCATCTATG	CTCTGCACGGGACAGCAAT	NM_011577.2
*XIAP*	CCATGTGTAGTGAAGAAGCCAGAT	GATCATCAGCCCCTGTGTAGTAG	NM_009688.3

**Table 2:** Cell proliferation activities of 6FC on Hep3B cells.

	Cell Number (×10^5^ per mL)					
	HGF (10 ng/mL)					
Hep3B	−	+	+	+	+	+
	Concentration of Compound (µM)					
Compounds	0	0	0.5	1	5	10
Control	1.90 ± 0.09	2.14 ± 0.05	2.18 ± 0.07	2.17 ± 1.53	2.15 ± 0.13	2.20 ± 0.27
Sm ^a^			2.27 ± 0.21	2.31 ± 0.13	2.33 ± 0.06 *	2.51 ± 0.12 *
6FC			2.41 ± 0.10 *	2.44 ± 0.06 *	2.82 ± 0.09 *^,#^	3.08 ± 0.13 *^,#^

Equal numbers of cells were treated 10 ng/mL HGF and indicated concentrations of 6FC. Cell growth was determined after 4 days by counting cell numbers. Data represent the mean ± s.d. of three independent experiments. In the table, “−” indicates without HGF treatment, and “+” indicates with HGF treatment. ^a^ Positive control. * *p* < 0.05 vs. control (DMSO alone). ^#^ *p* < 0.05 vs. positive control.

**Table 3:** Dose-dependency of cell proliferation activities of 6FC on Hep3B cells.

	Cell Number (×10^5^ per mL)				
	HGF (10 ng/mL)				
Hep3B	Day After Treatment (10 µM)				
Compounds	0	1	2	3	4
Control	0.50 ± 0.06	1.04 ± 0.22	1.32 ± 0.09	2.05 ± 0.08	2.23 ± 0.25
Sm ^a^		1.16 ± 0.20	1.52 ± 0.28	2.22 ± 0.03 *	2.71 ± 0.05 *
6FC		1.24 ± 0.12	2.32 ± 0.19 *	2.88 ± 0.28 *^,#^	3.05 ± 0.14 *^,#^

Equal number of cells were treated with 10 ng/mL HGF and 10 µM of 6FC. Cells were counted every 24 h. Data represent the mean ± s.d. of three independent experiments. ^a^ Positive control. * *p* < 0.05 vs. control (DMSO alone). ^#^ *p* < 0.05 vs. positive control.

## Data Availability

The data that support the findings of this study are available from the corresponding author upon reasonable request. Some data may not be made available because of privacy or ethical restrictions.

## Supplementary Materials

The following supporting information can be downloaded at: https://www.mdpi.com/article/10.3390/antiox14101210/s1, Figure S1: The chemical structure of 6FC; Figure S2: The HPLC analysis of the 70% ethanol extract of the stem bark of *C. ovata* (A) and 6FC (B). References cited: [12].

## Abbreviations

ALT, alanine aminotransferase; AP-1, activator protein-1; AST, aspartate aminotransferase; EGFR, epidermal growth factor receptor; HapG, haptoglobin; HGF, hepatocyte growth factor; IKK, IκB kinase; MAPK, mitogen-activated protein kinase; NF-κB, nuclear Factor kappa-light-chain-enhancer of activated B cells; PHx, Partial hepatectomy; SAA, serum amyloid A; Sm, silymarin; STAT3, signal transducer and activator of transcription 3; 6FC, 6-*O*-*trans*-feruloyl catalpol.
