# Supplementary material for: 6-O-trans-feruloyl Catalpol, a Natural Antioxidant from the Stem Bark of Catalpa ovata, Accelerates Liver Regeneration In Vivo via Activation of Hepatocyte Proliferation Signaling Pathways
Source: Antioxidants (Basel). 2025 Oct 6;14(10):1210. doi: 10.3390/antiox14101210 (PMC12562055; doi:10.3390/antiox14101210)
Supplement: Supplementary file 1 [file antioxidants-14-01210-s001.zip › antioxidants-3877828-supplementary.pdf]

Supporting Information

Figure S1 The chemical structure of 6FC.

Figure S2 The HPLC analysis of the 70% ethanol extract of the stem bark of *C. ovata* (A) and 6FC (B).

Figure S1 The chemical structure of 6FC.

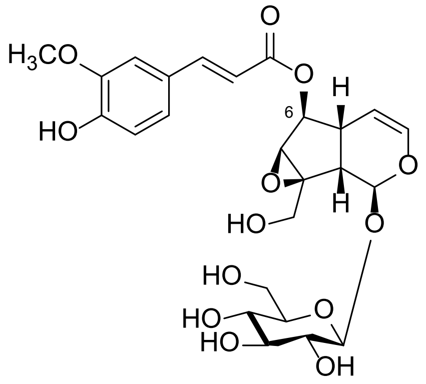

Figure S2 The HPLC analysis of the 70% ethanol extract of the stem bark of *C. ovata* (A) and 6FC (B).

(A)

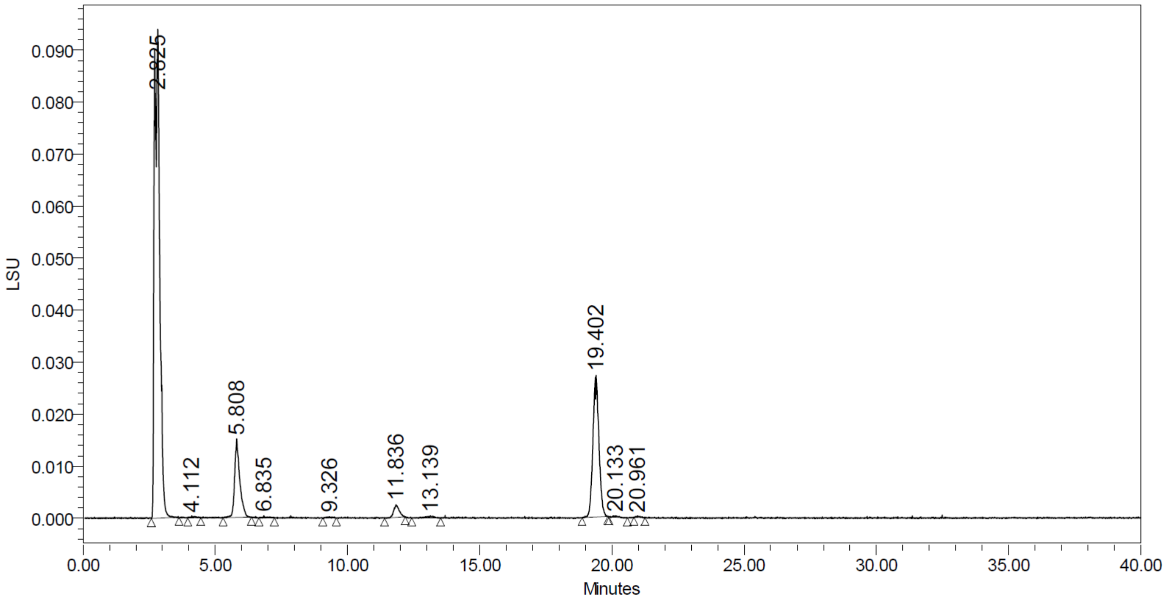

	RT	Area	% Area	Height
1	2.825	1330	66.15	94
2	4.112	4	0.18	0
3	5.808	201	10.00	15
4	6.835	2	0.10	0
5	9.326	2	0.10	0
6	11.836	35	1.76	2
7	13.139	7	0.37	0
8	19.402	424	21.07	27
9	20.133	4	0.18	0
10	20.961	2	0.10	0

(B)

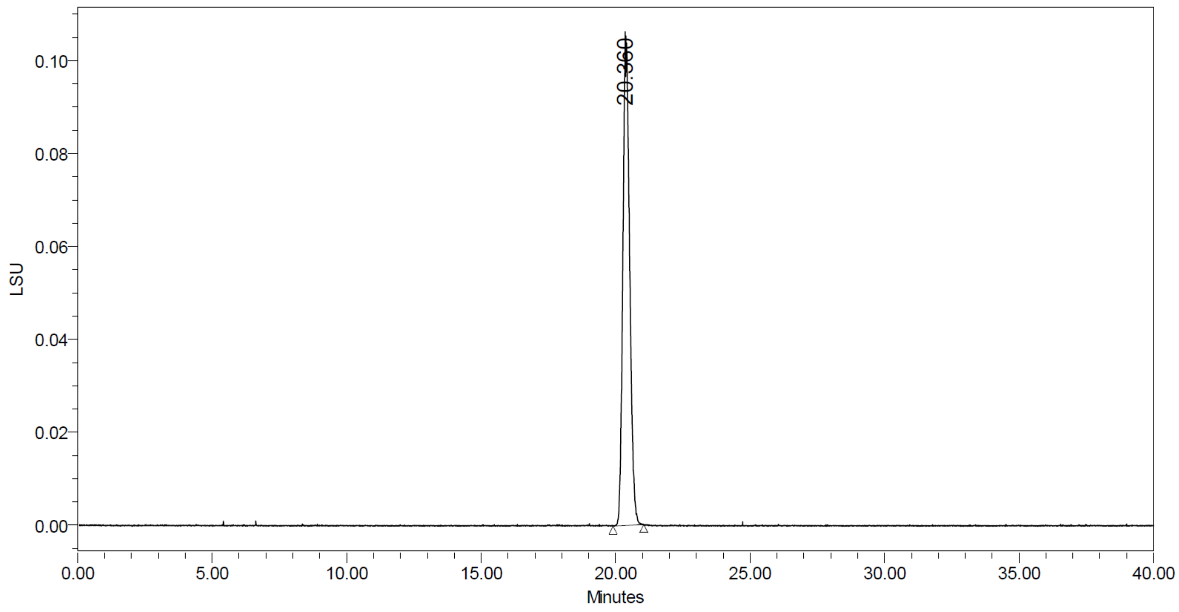

	RT	Area	% Area	Height
1	20.360	1835	100.00	106

The stem bark of *C. ovata* (100 g) in an ethanol-water mixture (7:3, 3 × 1 l) was sonicated at 25 °C for 30 min. The yielded extract was filtered, vacuum concentrated to afford an extract (33 g, approximately 33% yield). The 70% ethanol extract was accurately weighed to 5.00 mg and dissolved in 1 ml of methanol. 6FC was prepared as described previously (Kil, Kim, Kang, Chung, & Seo, 2017). HPLC analysis was conducted using Waters system (Waters Co., Milford, MA, USA) with a 2424 ELS detector and 1525 binary HPLC pump, and Waters Empower Pro (Waters Co., Milford, MA, USA) was used for data acquisition and integration. The samples were analyzed by reverse phase HPLC with a Phenomenex Luna 5μm C_{18} column (4.6 × 250 mm i.d., flow rate: 1 mL/min), using a gradient solvent system of methanol and water as follows: 0–30 min, 40-50% methanol; 30.1-40 min, 50-80% methanol. The drift tube temperature for ELSD was set at 60 ºC with the pressure of the nebulizing nitrogen gas of 50 psi. The ELSD

generates a signal in direct proportion to the quantity of an analyte present. Thus, the content of 6FC in the extract (retention time: 19.4 min in Figure S2A) was calculated 0.21 g/g (yield 21%). The presence of 6FC in the extract was confirmed by co-injection of the pure compound and the extract (data not shown). The purity of the pure compound was found to be greater than 99% (Figure S2B).

Reference

Kil, Y.S.; Kim, S.M.; Kang, U.; Chung, H.Y.; Seo, E.K. Peroxynitrite-Scavenging Glycosides from the Stem Bark of Catalpa ovata. *J. Nat. Prod.* **2017**, *80*, 2240–2251. https://doi.org/10.1021/acs.jnatprod.7b00139.
